# Hyperthyroidism is not a risk factor for subclinical bacteriuria in cats: A prospective cohort study

**DOI:** 10.1111/jvim.15769

**Published:** 2020-04-11

**Authors:** Mark E. Peterson, Alice Li, Peter Soboroff, Graham E. Bilbrough, Mark Rishniw

**Affiliations:** ^1^ Animal Endocrine Clinic New York New York USA; ^2^ College of Veterinary Medicine, Cornell University Ithaca New York USA; ^3^ New York Cat Hospital New York New York USA; ^4^ IDEXX Laboratories, Inc. Westbrook Maine USA; ^5^ Veterinary Information Network Davis California USA

**Keywords:** feline, hematuria, pyuria, subclinical bacteriuria, urinary tract infection, urine culture

## Abstract

**Background:**

Subclinical bacteriuria is defined as a positive bacterial urine culture in the absence of clinical evidence of urinary tract infection (UTI). Studies have reported that hyperthyroid cats have UTIs (mostly subclinical) with prevalence rates of 12%‐22%. Consequently, clinicians consider hyperthyroidism a risk factor for development of subclinical bacteriuria, and many recommend urine culture when evaluating hyperthyroid cats.

**Objectives:**

To compare the prevalence of subclinical bacteriuria (based on positive urine culture) in untreated hyperthyroid cats to that in euthyroid cats of similar age and sex.

**Animals:**

Three hundred and ninety‐three hyperthyroid cats presented for radioiodine treatment and 131 euthyroid cats (≥7 years of age) presented for routine examination. Cats with signs of lower urinary tract disease were excluded.

**Methods:**

Prospective cohort study. Both hyperthyroid and euthyroid cats had urine collected by cystocentesis for complete urinalysis and culture. Data pertaining to age, sex, body condition, and serum thyroxine and creatinine concentrations also were acquired. Logistic regression was performed to evaluate for potential risk factors for subclinical bacteriuria.

**Results:**

Hyperthyroid cats showed a low prevalence of subclinical bacteriuria (4.3%), which did not differ from that found in euthyroid cats (4.6%). Of the signalment factors evaluated, only female sex was a significant risk factor (odds ratio [OR], 6.9; *P* = .002). Furthermore, positive urine cultures were more likely in specimens with dilute urine concentration (<1.035), pyuria, or microscopic bacteriuria.

**Conclusions and Clinical Importance:**

Hyperthyroid cats are not at risk for subclinical bacteriuria. In the absence of lower urinary tract signs, no clinical benefit exists in routinely performing urine cultures when evaluating hyperthyroid cats.

**Abbreviations**
cTSH: canine TSH
IQR: interquartile range
SDMA: serum symmetric dimethylarginine
T_4_: thyroxine
TSH: thyroid stimulating hormone
UTI: urinary tract infection

## 1 INTRODUCTION

Subclinical bacteriuria (also called occult bacteriuria or asymptomatic bacteriuria) is defined as a positive bacterial urine culture in the absence of clinical evidence of urinary tract infection (UTI).^1^ Studies have recognized subclinical bacteriuria as a common clinical syndrome in humans, cats, and dogs.^1^, ^2^, ^3^, ^4^ Retrospective studies of hyperthyroid cats have reported a prevalence of UTI ranging from 12 to 22%,^5^, ^6^ and consisting mostly of subclinical bacteriuria. Consequently, clinicians consider hyperthyroidism a risk factor for development of UTI,^3^, ^7^ and many recommend urine culture as a part of the routine evaluation of hyperthyroid cats.^8^, ^9^

However, in our experience, the prevalence of subclinical bacteriuria in hyperthyroid cats appears to be much lower than previously reported. Furthermore, none of the reported studies^5^, ^6^, ^10^ suggesting hyperthyroidism as a risk factor for subclinical bacteriuria examined the prevalence of subclinical bacteriuria in a similarly aged, control population of euthyroid cats. Because age is a risk factor for subclinical bacteriuria,^3^, ^11^, ^12^ the effect of hyperthyroidism (95% of which occurs in cats >9 years old^13^) on development of subclinical bacteriuria requires an appropriate control group to eliminate the confounding effect of age.

Therefore, we sought to better determine the true prevalence of subclinical bacteriuria in cats with hyperthyroidism. To that end, we screened cats with hyperthyroidism for subclinical bacteriuria, and compared the prevalence of bacteriuria (based on positive urine culture) in these cats to a population of euthyroid cats of similar age and sex that presented for routine examination. Our secondary objectives were to investigate potential risk factors for subclinical bacteriuria (female sex, older age, presence of kidney disease) in our 2 prospectively collected populations of middle‐aged to older cats, as well as to evaluate urine sediment findings (eg, hematuria, pyuria, bacteriuria) as predictors of a positive urine cultures.

## 2 MATERIALS AND METHODS

### 2.1 Study design and selection of animals

#### 2.1.1 Hyperthyroid cats

All hyperthyroid cats referred to our clinic for treatment with radioiodine over the 22‐month period from January 2018 to October 2019 were evaluated for inclusion in this prospective cohort study. To be eligible for inclusion, hyperthyroid cats underwent a thorough evaluation that included review of past medical history, complete physical examination (including body weight, body, and muscle condition scoring),^14^ routine laboratory testing (CBC, serum biochemical profile, and complete urinalysis), urine culture, determination of serum thyroid hormone concentrations (total T_4_ and TSH),^15^, ^16^ and qualitative and quantitative thyroid scintigraphy.^17^ In cats previously treated with methimazole, the drug was discontinued ≥7 days before evaluation. None of these hyperthyroid cats had been treated recently with antibiotics. We excluded cats with signs of lower urinary tract disease, and those in which cystocentesis was not possible, either because of small bladder size or the fractious nature of the cat.

#### 2.1.2 Clinically normal, euthyroid cats

These cats were recruited as controls at time of visit for routine evaluation. To be enrolled in the study, cats had to be ≥7 years of age and considered healthy by their owners. None of these cats had any signs of lower urinary tract disease or had been recently treated with antibiotics, and all were normal on physical examination. These cats also were evaluated by routine laboratory testing (CBC, serum biochemical profile, and complete urinalysis) and urine culture, as well as serum total T_4_ and TSH concentrations to exclude hyperthyroidism.^15^, ^16^ Again, we excluded cats in which urine could not be collected by cystocentesis.

Ethics approval was obtained from the Institution's Animal Care and Use Committee (IACUC) before the study commenced. All owners provided informed consent.

### 2.2 Collection and processing of urine samples

Cystocentesis was performed without sedation on cats in lateral recumbency using manual palpation of the bladder and alcohol skin preparation.

Urine samples for urinalysis and culture (≥3 mL) were placed in appropriate sterile, plastic collection tubes, stored at 4°C, and transported to the laboratory (IDEXX Reference Laboratories, Westbrook, Maine, Reference Laboratory location, Manhattan, New York), where they remained refrigerated until being processed within 12 hours of collection. All procedures for routine urinalyses and culture were performed by trained laboratory technicians.

#### 2.2.1 Routine complete urinalysis procedures

For complete urinalysis, samples were allowed to warm to room temperature and then analyzed within 1 hour. Urine specific gravity was measured using a refractometer (Leica Vet 360, Misco Products Division, Cleveland, Ohio). Dipstick analysis was performed using Multistix 10 SG Reagent Strips on a Clinitek 500 Urine Chemistry Analyzer (Siemens Medical Solutions USA, Malvern, Pennsylvania).

For urine sediment evaluation, 60 μL of a well‐mixed, uncentrifuged urine sample were pipetted into 1 well of a 96‐well plate (Falcon tissue culture flat bottom well plate, REF 353075; Falcon, Corning, NY), and the sample was allowed to settle for 10 minutes.^18^ Using an inverted microscope (Olympus CKX41; Olympus Corporation of the Americas, Center Valley, Pennsylvania), images were captured under high power field (hpf) for evaluation. An active sediment was defined as pyuria (>5 white blood cells/hpf), hematuria (>10 red blood cells/hpf), or bacteriuria (any number). When the presence of bacteria was deemed questionable, a dried cytocentrifuge preparation was prepared and stained with modified Wright‐Giemsa stain to better identify bacteriuria.

#### 2.2.2 Bacterial culture procedures

Urine first was cultured semiquantitively. One microliter of urine was inoculated on each of 2 culture media (blood and MacConkey's agar) plates and incubated at 35°C for up to 20 hours. A positive result for quantitative urine culture was defined as any growth of bacteria in the cystocentesis sample. If culture positive, the bacterial species next were identified by use of matrix‐assisted laser desorption/ionization and time‐of‐flight (MALDI‐TOF) mass spectrometry (MALDI Biotyper; Bruker Scientific LLC, Billerica, Massachusetts).^19^, ^20^ Antimicrobial susceptibility then was determined by use of VITEK 2 automated system (Vitek 2 XL, bioMerieux Inc., Durham, North Carolina),^19^, ^20^ whereby the minimal inhibitory concentration (MIC) was determined and microorganisms categorized as susceptible, intermediate, or resistant according to Clinical and Laboratory Standards Institute (CLSI) guidelines.^21^

### 2.3 Data and statistical analyses

Data were assessed for normality using the D'Agostino‐Pearson test and by visual inspection of graphical plots.^22^ Data were not normally distributed. Therefore, all analyses used nonparametric tests.^23^

Results for continuous data (ie, age, body weight, urine specific gravity, and serum T_4_, TSH, creatinine, and SDMA concentrations) were expressed as median (interquartile range [IQR], 25th‐75th percentile), and results for qualitative data are expressed as ratio (breed, sex) or number (%) of cats (prevalence of positive urine cultures). Continuous variables were compared between groups using the Mann‐Whitney *U* test. Categorical variables were compared among groups using the chi‐square test or Fisher's exact test, where appropriate.

To evaluate potential risk factors for subclinical bacteriuria, logistic regression^24^ was performed using urine culture results (positive versus sterile) as the dependent variable, and the following as independent variables: group (hyperthyroid versus euthyroid), age (years), age stage (mature [7‐10 years], senior [11‐14 years], or geriatric [≥15 years]),^25^ sex, body condition score (underweight, ideal weight, or overweight), and presence of azotemic kidney disease (defined as serum creatinine concentration >2.1 mg/dL and urine specific gravity <1.035).^15^ To select variables that best explained the probability of a cat being culture‐positive, we used a backward stepwise approach. Variables associated with a cat being culture‐positive at an alpha level <.2 were entered into the model. The significance of each explanatory variable was tested using the Wald test. Biologically plausible, multiplicative 2‐way interactions between the remaining variables were assessed for significance. Results of the final model are reported in terms of adjusted odds ratios (OR) with 95% confidence intervals (95% CI) for each explanatory variable.

A separate logistic regression similarly was performed for the complete urinalysis and sediment findings, again using urine culture results (positive versus sterile) as the dependent variable, and urine pH, urine specific gravity (<1.035 or ≥ 1.035), pyrexia, hematuria, and bacteriuria on urine sediment evaluation as the independent variables.

For all analyses, statistical significance was defined as *P* ≤ .05. All statistical analyses were performed using proprietary statistical software (GraphPad Prism, version 7.0; GraphPad Software, La Jolla, California; MedCalc, version 17.6, MedCalc Software, bvnv, Ostend, Belgium).

## 3 RESULTS

### 3.1 Signalment of cats

#### 3.1.1 Cats with hyperthyroidism

During the 24‐month study period, we evaluated 441 hyperthyroid cats for enrollment. Of these, 48 were excluded because of clinical signs of lower urinary disease (n = 3) or inability to collect urine by cystocentesis without sedation (n = 45). Thus, we included 393 hyperthyroid cats in our analysis.

The hyperthyroid cats ranged in age from 6 to 20 years (median, 12.0 years; Table 1). Based on their age stage, 88 (22.4%) of these cats were mature (7‐10 years), 232 (59%) were senior (11‐14 years), and 73 (18.6%) were geriatric (≥15 years).^25^ Breeds included domestic longhair and shorthair (341 cats), Maine Coon (15 cats), Siamese (13 cats), Bombay (4 cats), Bengal (3 cats), Burmese (3 cats), American Shorthair (3 cats), Scottish Fold (2 cats), Ragdoll (2 cats), Persian (2 cats), Abyssinian, Himalayan, Norwegian Forest Cat, Russian Blue, and Tonkinese (1 cat each). Of these, 204 were female (51.9%) and 189 were male; all had been neutered.

**TABLE 1:** Comparison of signalment, serum thyroid and renal function, and prevalence of positive urine culture results (subclinical bacteriuria) in hyperthyroid and euthyroid cats

Variable	Hyperthyroid (393)	Euthyroid (131)	*P* value
Age (years)	12 (11–14)	12 (10‐14)	.99^a^
Breed (mixed:pure breed ratio)	341:52 (6.56)	110:21 (5.24)	.47^b^
Sex (female:male ratio)	204:189 (1.08)	67:64 (1.05)	.92^b^
Body weight (kg)	4.2 (3.5‐5.0)	4.9 (4.1‐5.7)	<.001^a^
Serum T_4_ (μg/dL)	8.8 (6.6‐12.0)	2.0 (1.8‐2.5)	<.001^a^
Serum TSH (ng/mL)	0.02 (0.02‐0.02)	0.07 (0.04‐0.11)	<.001^a^
Serum creatinine (mg/dL)	1.1 (0.9‐1.4)	1.6 (1.4‐1.9)	<.001^a^
Serum SDMA (μg/dL)	10 (9‐13)	12 (10‐14)	.01^a^
Urine specific gravity	1.029 (1.019‐1.043)	1.034 (1.019‐1.046)	.06^a^
Positive urine culture (subclinical bacteriuria)	17 (4.3%)	6 (4.6%)	.99^b^

*Note:* All continuous data (age, body weight, serum T_4_, TSH, creatinine, SDMA, and urine specific gravity) are expressed as median (IQR). All qualitative data are expressed as ratio (breed, sex) or number (%) of cats (prevalence of positive urine cultures). Reference intervals: T_4_ = 0.9‐3.8 μg/dL; TSH = <0.03‐0.3 ng/mL; creatinine = 0.9‐2.1 mg/dL; SDMA = 0‐14 μg/dL.

a Mann‐Whitney *U* test.

b Fisher's exact test.

Body weights of the hyperthyroid cats ranged from 1.6 to 7.7 kg (median, 4.2 kg; Table 1); 100 (25.4%) cats were considered underweight, 235 (59.8%) had an ideal body condition score, and 58 (14.8%) were considered overweight.

Two hundred and seven cats (52.7%) had not received methimazole treatment, and 186 (47.3%) cats had been treated with methimazole for periods of 1 week to 4.5 years (median, 60 days; IQR, 45‐300 days). In all of the methimazole‐treated cats, the drug was discontinued ≥1 week (median, 7 days; IQR, 7‐15 days; range, 7‐150 days) before evaluation. No signalment variables differed between the cats that had been treated with methimazole and those that had not (Table 2).

**TABLE 2:** Comparison of signalment, serum thyroid and renal function, and prevalence of positive urine culture results (subclinical bacteriuria) in hyperthyroid cats subdivided into methimazole naïve and methimazole‐treated groups

Variable	No methimazole (207)	Methimazole (186)	*P* value
Age (years)	13 (11‐14)	12 (11‐14)	.38^a^
Breed (mixed:pure breed ratio)	179:28 (6.57)	161:25 (6.44)	.99^b^
Sex (female:male ratio)	112:95 (1.18)	92:94 (0.98)	.37^b^
Body weight (kg)	4.2 (3.6‐5.0)	4.2 (3.5‐5.0)	.94^a^
Serum T_4_ (μg/dL)	8.3 (6.6‐11.1)	10.1 (6.6‐14.3)	.001^a^
Serum TSH (ng/mL)	0.02	0.02	.93^a^
Serum creatinine (mg/dL)	1.1 (0.8‐1.4)	1.1 (0.9‐1.4)	.38^a^
Serum SDMA (μg/dL)	10 (9‐12)	11 (9‐13)	.47^a^
Urine specific gravity	1.030 (1.019‐1.042)	1.027 (1.019‐1.043)	.68^a^
Positive urine culture (subclinical bacteriuria)	8 (3.9%)	9 (4.8%)	.81^b^

*Note:* All continuous data (age, body weight, serum T_4_, TSH, creatinine, SDMA, and urine specific gravity) expressed as median (IQR). All qualitative data expressed as ratio (breed, sex) or number (%) of cats (prevalence of positive urine cultures).

a Mann‐Whitney *U* test.

b Fisher's exact test.

#### 3.1.2 Euthyroid cats

We evaluated 143 clinically normal cats as controls at the time of visit for a routine evaluation. Of these, we could not collect urine by cystocentesis in 12 cats, either because of small bladder size or the nature of the cat. Thus, we included 131 apparently healthy cats in our analysis.

The euthyroid cats ranged in age from 7 to 18 years (median, 12.0 years), similar to the hyperthyroid cats (Table 1). Based on their age stage, 33 cats (25.2%) were mature, 69 (52.7%) were senior, and 29 (22.1%) were geriatric. These euthyroid cats did not differ from the hyperthyroid cats in age (Table 1) or in distribution of age stages (*P* = .50).

Breeds included domestic longhair and shorthair (110 cats), Maine Coon (3 cats), Persian (4 cats), Siamese (3 cats), Tonkinese (2 cats), American Shorthair, Balinese, British Shorthair, Egyptian Mau, Himalayan, Japanese Bobtail, Ocicat, Rag doll, and Russian Blue (1 cat each). Of these cats, 67 (51.2%) were female and 64 were male; all had been neutered. These euthyroid cats did not differ from the hyperthyroid cats in breed or sex distribution (Table 1).

Body weights of the 131 euthyroid cats ranged from 2.8 to 9.9 kg (median, 4.9 kg; Table 1); 3 (2.3%) of these cats were considered underweight, 82 (62.6%) had an ideal body condition score, and 46 (35.1%) were considered overweight. Euthyroid cats were heavier than hyperthyroid cats (Table 1). Similarly, the euthyroid cats were less likely to be underweight and more likely to be overweight, as compared to the hyperthyroid cats (*P* < .001).

### 3.2 Thyroid and renal function testing

The hyperthyroid cats had high serum T_4_ concentrations together with undetectable serum TSH concentrations, both of which differed from the euthyroid cats (Table 1). Serum T_4_ concentrations of the euthyroid cats fell within reference intervals, and 102 (77.9%) cats had measurable (≥0.03 ng/mL) serum TSH concentrations. Serum T_4_ concentrations in the cats previously treated with methimazole (off drug for ≥7 days) were higher than in the cats that had not received methimazole (*P* = .001; Table 2). Serum TSH concentrations did not differ between the cats that had been treated with methimazole and those that had not (*P* = .93; Table 2).

Hyperthyroid cats had lower serum concentrations of both creatinine and SDMA than did euthyroid cats (Table 1). Urine specific gravity, however, did not differ between groups, with 71 (54.2%) of the euthyroid cats and 233 (59.3%) of the hyperthyroid cats having urine specific gravity <1.035 (Table 1). Serum creatinine concentrations, SDMA concentrations, and urine specific gravity did not differ between the cats that had been treated with methimazole and those had not (Table 2).

We identified azotemia (serum creatinine concentration range, 2.2‐2.7 mg/dL) in 12 (3.1%) of the untreated hyperthyroid cats. Within 6 months of radioiodine treatment, an additional 43 cats (10.9%) developed azotemia (serum creatinine concentration range, 2.2‐3.8 mg/dL) as their hyperthyroidism resolved. Similarly, we identified azotemia in 13 (9.9%) of the euthyroid cats (range of serum creatinine concentration, 2.2‐3.2 mg/dL). The proportion of hyperthyroid cats with azotemic kidney disease (57 cats; 14.5%) was similar to the proportion of azotemic kidney disease (13 cats; 9.9%) in the euthyroid cats (*P* = .24).

### 3.3 Urine culture results

Hyperthyroid cats had a prevalence of subclinical bacteriuria of 4.3% (17/393), which did not differ from that found in euthyroid cats (4.6%; 6/131; Table 1). Likewise, hyperthyroid cats treated with methimazole had a similar prevalence of subclinical bacteriuria to those that had not received methimazole (Table 2).

Ages of the cats with subclinical bacteriuria (hyperthyroid or euthyroid) did not differ from the ages of cats without subclinical bacteriuria (Table 3). More female cats (20 of the combined 23 hyperthyroid and euthyroid cats) had subclinical bacteriuria than did male cats (*P* = .001; Table 3).

**TABLE 3:** Comparison of signalment, serum thyroid, and renal function, and urine sediment in hyperthyroid and euthyroid cats, subdivided into cats with negative and positive urine culture results

Finding	Hyperthyroid culture negative (376)	Hyperthyroid culture positive (17)	Euthyroid culture negative (125)	Euthyroid culture positive (6)
Age (years)	12 (11‐14)	13 (12‐14.5)	12 (10‐14)	12 (11‐14)
Sex (female:male)	189:187 (1.01)	15:2^a^ (7.5)	62:63 (0.98)	5:1 (5.0)
Body weight (kg)	4.2 (3.6‐5.0)	4.1 (3.2‐5.0)	4.9 (4.1‐5.7)	5.1 (3.8‐6.5)
Serum T_4_ (μg/dL)	8.6 (6.5‐12.2)	10.2 (8.8‐11.7)	2.0 (1.8‐2.5)	2.0 (1.6‐2.5)
Serum creatinine (mg/dL)	1.1 (0.9‐1.4)	1.1 (0.9‐1.4)	1.6 (1.4‐1.9)	1.5 (1.5‐1.7)
Urine specific gravity	1.030 (1.019‐1.042)	1.020^b^ (1.015‐1.040)	1.036 (1.020‐1.047)	1.023 (1.016‐1.040)
Hematuria (>10 RBC/hpf)	71 (18.9%)	5 (29.4%)	25 (20.0%)	2 (33.3%)
Pyuria (>5 WBC/hpf)	10 (2.7%)	3^a^ (17.6%)	4 (3.2%)	3^a^ (50%)
Bacteria sediment (any number)	10 (2.7%)	9^a^ (52.9%)	2 (1.6%)	2^a^ (33.3%)
Active sediment (hematuria, pyuria, or bacteriuria)	88 (23.4%)	12^a^ (70.6%)	29 (23.2%)	4^a^ (66.6%)

*Note:* All continuous data expressed as median (IQR). Qualitative data (sex) expressed as ratio.

a *P* < .01 (Fisher's exact test) compared to cats without subclinical bacteriuria.

b *P* < .05 (Mann‐Whitney *U* test) compared to cats without subclinical bacteriuria.

In the euthyroid cats, similar serum T_4_ concentrations were found in the cats that had positive or negative urine culture results. Hyperthyroid cats with subclinical bacteriuria had serum T_4_ concentrations that were slightly higher but not significantly different from hyperthyroid cats with negative cultures (*P* = .86; Table 3).

None of the 6 euthyroid cats and only 2 (11.8%) of the 17 hyperthyroid cats with subclinical bacteriuria were azotemic. Accordingly, serum creatinine concentrations in the cats with subclinical bacteriuria (hyperthyroid or euthyroid) did not differ from those of the cats with negative urine cultures (Table 3).

Hyperthyroid cats, but not euthyroid cats with subclinical bacteriuria had lower urine specific gravity than did corresponding cats with negative cultures (*P* = .05 for hyperthyroid cats; *P* = .20 for euthyroid cats; Table 3). Because thyroid status was not associated with probability of subclinical bacteriuria, we grouped all hyperthyroid and euthyroid cats and found that the 23 cats with subclinical bacteriuria had lower urine specific gravity than did the 501 cats with negative cultures (*P* = .02). Of the 6 euthyroid cats and 17 hyperthyroid cats with subclinical bacteriuria, urine specific gravity was <1.035 in 4 (66.7%) and 12 (70.5%) cats, respectively.

The prevalence of pyuria, bacteriuria, and active sediment were higher in both hyperthyroid and euthyroid cats with positive urine culture results (Table 3). However, the likelihood of microscopic hematuria was similar for cats with positive and negative urine culture results (Table 3).

### 3.4 Organisms

Of the 17 hyperthyroid cats with bacterial growth, the most common isolates were *Escherichia coli* and *Enterococcus faecalis* (Table 4). Two hyperthyroid cats had a mixed polymicrobial infection with *E. coli* isolated with either *E. faecalis* or *Staphylococcus felis*.

**TABLE 4:** Urine culture results and degree of growth for 6 euthyroid cats and 17 hyperthyroid cats

Group	Bacteria isolated	Heavy growth >100 000 CFU/mL	Moderate growth 10 000‐100 000 CFU/mL	Light growth <10 000 CFU/mL	Total no. with growth
Hyperthyroid					
	*Escherichia coli*	2	4	2	8
	*Enterococcus faecalis* *	4	2		6
	*Bacillus species* *			1	1
	*Corynebacterium species* *		1		1
	*Klesiella species*	1			1
	*Staphylococcus felis* *		1		1
	*Staphylococcus haemolyticus* *			1	1
	Total isolates				19*
Euthyroid					
	*E. faecalis* *	3			3
	*S. felis* *	2			2
	*Proteus mirabilis*		1		1
	Total isolates				6

a Two hyperthyroid cats had a *E. coli* isolated in combination with either *E. faecalis* or *S. felis*.

In the 6 euthyroid cats, the most common isolates were *E. faecalis* and *S. felis* (Table 4). In contrast to the hyperthyroid cats, *E. coli* was not isolated from any of the euthyroid cats.

### 3.5 Predictors of subclinical bacteriuria

Hyperthyroidism did not increase the risk for subclinical bacteriuria (Table 5). Among all cats, risk of subclinical bacteriuria was not associated with age, age stage, body condition, or azotemic kidney disease (Table 5). However, female cats had a significantly increased risk of subclinical bacteriuria compared to male cats (Table 5).

**TABLE 5:** Odds ratios for the potential risk factors for subclinical bacteriuria (positive urine culture) for the 393 hyperthyroid and 131 euthyroid cats

Factor	Odds ratio	95% confidence interval (CI)	*P* value
Thyroid status (hyperthyroid, euthyroid)	0.9	0.4‐2.6	.90
Age	1.1	0.9‐1.5	.46
Geriatric age stage	1.1	0.3‐4.4	.93
Female sex	6.9	2.3‐29.9	.002
Underweight	0.7	0.2‐2.0	.56
Overweight	0.8	0.2‐2.5	.70
Azotemic kidney disease	1.0	0.2‐3.1	.97
Dilute urine specific gravity (<1.035)	3.5	1.1‐12.9	.04
Urine pH	1.0	0.5‐2.2	.92
Hematuria (>10 RBC/hpf)	1.8	0.6‐5.4	.29
Pyuria (>5 WBC/hpf)	6.9	1.3‐28.1	.01
Bacteriuria in sediment (any number)	31.4	10.1‐98.7	<.001

Among all cats, no significant association existed between urine pH or hematuria and the presence of a positive culture regardless of thyroid category (Table 5). In contrast, the presence of dilute urine concentration (<1.035), pyuria, and bacteriuria all were associated with increased risk for subclinical bacteriuria (Table 5).

### 3.6 Follow‐up of cats with subclinical bacteriuria

Of the 23 cats with subclinical bacteriuria (17 hyperthyroid and 6 euthyroid), 2 of the hyperthyroid cats were lost to follow‐up shortly after initial evaluation and treatment with radioiodine. The remaining 21 cats with subclinical bacteriuria were followed up for 4 to 24.5 months (median, 12.5 months; IQR, 6.3‐19.6 months). None of these cats developed signs of urinary tract disease over that follow‐up period. Follow‐up urine cultures were performed in 4 of the 21 cats (19%), and all remained urine culture positive.

## 4 DISCUSSION

The results of our prospective study indicate that hyperthyroid cats have a low prevalence (<5%) of subclinical bacteriuria, nearly identical to that found in a cohort of euthyroid cats of similar age and sex presented for routine examination. This finding is in marked contrast to previous retrospective studies that reported a relatively high prevalence of UTI in hyperthyroid cats (12%‐22%), findings that have led to the widespread belief that hyperthyroid cats are at risk for developing subclinical bacteriuria.^3^, ^8^, ^9^, ^10^ Our findings agree with other recent studies of mixed populations of cats, which failed to identify an increased risk of subclinical bacteriuria in much smaller cohorts of cats with hyperthyroidism.^26^, ^27^

Because urine culture was performed in all hyperthyroid cats evaluated for treatment during the 22‐month study period (only cats in which urine could not be collected by cystocentesis were excluded), our findings likely reflect the true prevalence of positive urine cultures in hyperthyroid cats not showing any clinical signs of UTI. Our results confirm that hyperthyroid cats are not at increased risk for subclinical bacteriuria. Therefore, urine culture should not be included as part of routine evaluation of these cats, unless clinical signs of lower urinary tract disease are present.

The low prevalence of subclinical bacteriuria in our hyperthyroid cats is similar to that reported in human patients with hyperthyroidism, in which cystitis or asymptomatic bacteriuria is very rare.^28^, ^29^, ^30^ In fact, reviews of asymptomatic bacteriuria in humans do not mention thyroid disease as a possible risk factor.^2^, ^31^

Common issues with study design of the past reports^5^, ^6^, ^10^ that promoted hyperthyroidism as a risk factor for subclinical bacteriuria in cats include their retrospective nature and lack of an appropriate control population of euthyroid cats. If age‐ and sex‐matched controls had been included in these studies, it is possible that a similar prevalence of subclinical bacteriuria also might have been found in those euthyroid cats. In addition, approximately 30% of the hyperthyroid cats in those retrospective studies had signs of cystitis (not subclinical bacteriuria), leading to selection of these cats for culture.^5^, ^6^, ^7^, ^10^ In our study, we prospectively evaluated over 400 hyperthyroid cats that presented to our clinic and excluded only 3 cats (<1%) because of clinical signs of lower urinary tract disease. Therefore, in contrast to early studies, urine culture was performed for the purpose of our study and not simply at the discretion of the clinician.

Some clinicians might have questioned whether methimazole treatment, by lowering high serum thyroid hormone concentrations to euthyroid concentrations, could decrease the prevalence of positive urine cultures in that subgroup of treated cats. Alternatively, it is possible that methimazole has a direct protective effect against development of subclinical bacteriuria. Our results do not support these hypotheses because we found a virtually identical prevalence of subclinical bacteriuria in hyperthyroid cats that had been treated with methimazole and cats that had not been treated with methimazole (Table 2). In addition, no difference in prevalence existed between the euthyroid control cats and the hyperthyroid cats (treated or not treated with methimazole). Serum T_4_ concentrations in the cats that had been treated with methimazole (ie, the drug was discontinued ≥7 days before evaluation) were higher than in those that had not been treated with methimazole. This finding was not unexpected because hyperthyroidism is progressive in cats,^32^, ^33^ and treating with methimazole over weeks to months allows the underlying thyroid disease to become more severe. Nevertheless, prior treatment with methimazole had no effect on development of subclinical bacteriuria in our study.

A wide range of prevalence rates for subclinical bacteriuria has been reported in cats, varying from low rates of <1.0%‐3.3%,^12^, ^34^, ^35^ to intermediate rates of 6.1, 7.9, and 10%‐13%,^26^, ^27^, ^36^ to a high rate of 28.8%.^11^ The main difference in these studies was the age of the cats, which tended to be younger in the studies with lower prevalence rates^12^, ^34^, ^35^ and older in the studies with prevalence rates >6%.^11^, ^26^, ^36^ Despite the fact that our euthyroid cats were older (median age, 12 years; range, 7‐20 years), these cats had a prevalence (<5%) of subclinical bacteriuria that was not nearly as high as reported in some studies.^11^, ^36^ Although the reason for these differences in prevalence is not clear, most of our euthyroid cats had no evidence for concurrent disease; all were considered clinically normal by their owners and only a few had biochemical evidence of any comorbidities (eg, mild renal disease). In contrast, some of the previous studies included many cats with concurrent disease,^11^ which might have increased the prevalence of subclinical bacteriuria.

We found females to be at higher risk for developing subclinical bacteriuria than males (OR, 6.9; Table 5), regardless of thyroid status, a finding that is in accord with other studies in cats.^26^, ^36^ However, we found that neither age nor age stage posed an increased risk for subclinical bacteriuria, similar to a large study of 179 middle‐aged to elderly cats,^27^ but in contrast to others in which older age was a risk factor.^26^, ^36^ A possible explanation for these differences could be the selection of middle‐aged to older cats in our study (no cats <7 years of age were included), which likely affected the association between increasing age and subclinical bacteriuria.

Some investigators have suggested that being either underweight or overweight also increases risk for subclinical bacteriuria in cats,^6^, ^26^, ^27^, ^37^ but we failed to observe this association. Likewise, we did not find azotemic renal disease to increase risk, similar to findings in some^27^, ^36^ but not all studies.^26^

Although azotemia was not predictive of subclinical bacteriuria, evidence of kidney disease in the form of dilute urine specific gravity (<1.035) did increase the risk, similar to findings in some,^5^, ^26^, ^37^ but not all studies.^6^, ^11^, ^36^ In addition, both pyuria and bacteriuria were predictors for subclinical bacteriuria, similar to findings in other studies.^6^, ^11^, ^26^ Although hematuria was common, it was not a predictor for subclinical bacteriuria in our study. Because cystitis can lead to the presence RBC in urine sediment, hematuria might be expected in cats with subclinical bacteriuria.^3^, ^26^ However, cystocentesis itself can induce transient microscopic hematuria indistinguishable from pathological hematuria,^38^, ^39^ and the number of RBCs in the sediment cannot be used as a reliable predictor of a positive urine culture.

Currently, insufficient evidence exists as to whether subclinical bacteriuria in cats should be treated or not. In human patients, standard of care is not to treat asymptomatic bacteriuria with antimicrobials, unless the patient is pregnant, or having invasive urinary procedures performed.^2^, ^31^, ^40^, ^41^ Most untreated patients with asymptomatic bacteriuria never develop symptomatic UTI and do not have adverse consequences. Treatment of humans with asymptomatic bacteriuria does not decrease the frequency of future episodes of bacterial cystitis, pyelonephritis, renal impairment, or survival. By promoting development of antimicrobial resistance, antibiotic treatment may increase the risk of clinical UTI.^2^, ^40^, ^41^, ^42^

Direct comparisons between human and veterinary medicine can be problematic, and future prospective studies comparing outcomes with and without antimicrobial treatment in cats with subclinical bacteriuria are needed. Current veterinary guidelines^1^ on management of UTI in cats recommend that, with some exceptions (eg, pyelonephritis), clinicians should not treat subclinical bacteriuria. In support of those guidelines, subclinical cats left untreated (including those with pyuria and bacteriuria) do not appear to have worse outcomes, even in the presence of comorbidities such as diabetes mellitus or chronic kidney disease.^10^, ^43^ In our study, none of the affected hyperthyroid cats were treated with antimicrobials, and our study protocol did not require follow‐up for repeat urine culture. However, none of the cats have displayed any signs of lower urinary tract disease since the time of urine culture and radioiodine treatment (4‐ to 24.5‐month follow‐up period). Our findings further support the current guidelines^1^ that clinicians refrain from treating subclinical bacteriuria in cats (regardless of thyroid status) with rare exceptions.

In conclusion, results of our prospective cohort study indicate that hyperthyroid cats are not at increased risk for developing subclinical bacteriuria. Routine urine culture in hyperthyroid cats incurs an unnecessary expense for owners and need not be included in the routine diagnostic evaluation of hyperthyroid cats unless clinical signs of lower urinary tract disease are present. One unanswered question is whether subclinical bacteriuria resolves or persists after treatment of hyperthyroidism in these cats. Further prospective studies designed to clarify the long‐term outcome of treated hyperthyroid cats are warranted.

## CONFLICT OF INTEREST DECLARATION

Graham E. Bilbrough is an employee of IDEXX Laboratories, where the urinalysis and urine cultures were done.

## OFF‐LABEL ANTIMICROBIAL DECLARATION

Authors declare no off‐label use of antimicrobials.

## INSTITUTIONAL ANIMAL CARE AND USE COMMITTEE (IACUC) OR OTHER APPROVAL DECLARATION

Authors declare no IACUC or other approval was needed.

## HUMAN ETHICS APPROVAL DECLARATION

Authors declare human ethics approval was not needed for this study.
